# Plasma acylcarnitines could predict prognosis and evaluate treatment of IgA nephropathy

**DOI:** 10.1186/s12986-018-0328-1

**Published:** 2019-01-08

**Authors:** Fang-Ying Xia, Li Zhu, Chao Xu, Qing-Qing Wu, Wan-Jia Chen, Rong Zeng, Yue-Yi Deng

**Affiliations:** 1 Department of Nephrology, Longhua Hospital, Shanghai University of Traditional Chinese Medicine, 25 South Wanping Road, Shanghai, 200032 China; 2 CAS Key Laboratory of Systems Biology, Institute of Biochemistry and Cell Biology, Shanghai Institutes for Biological Sciences, Chinese Academy of Sciences, 320 Yueyang Road, Shanghai, 200031 China

**Keywords:** Plasma acylcarnitine, IgA nephropathy, Traditional Chinese medicine, Biomarker, Prognosis and treatment responses

## Abstract

**Background:**

Effective evaluation or prediction of therapy response could be helpful for treatment of chronic kidney disease (CKD), which may rely on accurate biomarkers. Acylcarnitines are involved with lipid metabolism and mitochondrial function. The relation of acylcarnitines with treatment response in patients with CKD is unknown. The purpose of this study is to investigate the association of plasma acylcarnitines with renal function and its alteration by intervention in patients with IgA nephropathy (IgAN).

**Methods:**

A retrospective study was performed in 81 IgAN patients with treatment by traditional Chinese medicine (TCM). Multivariate linear regression analyses were performed to identify the association of acylcarnitines with baseline estimated glomerular filtration rate (eGFR) and eGFR changes after treatment.

**Results:**

Twenty-seven acylcarnitines were measured at baseline and after 1-year TCM intervention. Certain short-chain and median-chain acylcarnitines were independently associated with baseline eGFR and eGFR alterations after 1 year treatment. Particularly, patients with high C5:1(β = − 0.42), C8:1(β = − 0.49), C3DC(β = − 0.5), C10:1(β = − 0.36) and C5DC(β = − 0.64)at baseline would have worse prognosis and treatment response. Moreover, certain acylcarnitines could be changed along with the eGFR alteration after 1-year TCM treatment.

**Conclusions:**

The findings indicate a significant association between plasma acylcarnitines with prognosis and treatment responses in patients with IgAN, which suggest its role as a potential penal of biomarker for IgAN.

**Electronic supplementary material:**

The online version of this article (10.1186/s12986-018-0328-1) contains supplementary material, which is available to authorized users.

## Background

IgA nephropathy (IgAN) is the most common primary kidney disease with complicated pathogenesis, thus the progression of IgAN would be varying due to multiple factors. Though a few therapies could be selected, the responses to specific treatment are often uncertain due to the lack of efficient standard to evaluate. Generally, the baseline creatinine, estimated glomerular filtration rate (eGFR), proteinuria, hypertension etc. could be used as indicators to predict the prognosis [1, 2]. However, these physiological parameters may be varied and not sensitive enough to predict prognosis and evaluate therapy. The molecular markers would be more sensitive and accurate to evaluate the physiological and pathogenic status [3, 4]. The immunological proteins,complement components and cytokines have been investigated to be used as biomarkers for IgA diagnosis and prognosis. Currently, the omics-based technologies provide opportunity to systematic discovery of disease-related characteristics, which could be used for diagnosis and prognosis [5]. Acylcarnitines are associated with lipid metabolism and mitochondrial function, which would be changed with the decline of eGRF and might be biomarkers for chronic kidney disease (CKD) [6, 7]. However, the possibility of acylcarnitines as indicator of treatment response is not clear. The efficient treatment of CKD could improve mitochondrial function and metabolism, thus we hypothesized the association of acylcarnitine with treatment. In this work, we measured the acylcarnitines in IgA patients receiving traditional Chinese medicine (TCM) based therapy. The acylcarnitines before and after TCM-based treatment were quantified. The changes of acylcarnitines within observation period may reflect the treatment responses.

## Materials and methods

### Patient cohort

We reviewed records of IgAN database in Longhua hospital. Patients with overlapping histopathologic diagnosis or a concomitant clinical diagnosis of diabetes, malignancy, and chronic rheumatologic and Hepatitis B virus (HBV) infection, were excluded. Study participants were 81 patients with CKD stage 1–4, a renal diagnosis of IgA nephropathy by biopsy. All of the patients received treatment covering follow-up periods according to TCM-based therapy [8, 9], partially plus oral prednisone less than 8 months. Participants with hypertension used ACEI/ARB antihypertensive drugs. The major TCM include edastragalusmembranaceus, rhizomapolygonatipreparata, eucommiaulmoides, *Prunella vulgaris*, tribulusterrestris, epimedium, poriacocos, fried silkworm, salvia miltiorrhiza. Mean time of treatment was 12 months (range, 10–15 months). The plasma samples before (T1) and after (T2) treatments were collected. Signed informed consents from participants were obtained, and the study protocol has been approved by ethical committee of Longhua Hospital. The collection procedures were performed in accordance with the relevant guidelines and regulations.

### Outcome definition

eGFR was usually used to estimate the renal function, and many prospective studies use a reduction in eGFR as one of the endpoints of the study. For example, Rauen T et al. used two end points: full clinical remission and a decrease in the estimated GFR (eGFR) of at least 15 ml per minute per 1.73 m2 of body-surface area after 3 years of follow-up [10]. In this study, the primary outcome was eGFR and eGFR change, defined as the ratio of treatment level/baseline level of eGFR and we set 20% eGFR increase or decrease as the cut-point to judge the effect of therapy in receiver operating characteristic (ROC) curve analyses.

eGFR was calculated using the Modification of Diet in Renal Disease (MDRD) Study Equation for Chinese with minor modification [11]: eGFR (mL/min/1.73m^2^) = a × (Pcr/b)^c^ × (0.993)^age^ (a = 141, b = 79.56, c = − 0.411, if male and Pcr ≤ 79.56; a = 141, b = 79.56, c = − 1.209, if male and Pcr>79.56; a = 144, b = 61.88, c = − 0.329, if female and Pcr ≤ 61.88; a = 144, b = 61.88, c = − 1.209, if female and Pcr>61.88).

### Blood collection

Blood samples were collected by EDTA tubes, and centrifuged at 3000 rpm/min for 15 min at 4 °C. The supernatant was collected and stored in sterile 1.5 mL microcentrifuge tubes at − 80 °C until further analysis [9].

### Preparation of internal standard solutions and calibrations curves

The measurement of acylcarnitines was according to reported methods with modifications [12]. A combined standard solution was prepared by mixing respective stock solution to following concentration and stored at − 20 °C: 277.56 μmol/l of C0, 114.12 μmol/l of C2, 30.24 μmol/l each of C3, C3DC, 14.4 μmol/l each of C4, C4OH, C5, C5DC, C5OH, 17.76 μmol/l each of C6, C8, C10, C10:1, C12, 13.32 μmol/l each of C14:1, C14, and 28.08 μmol/l each of C16OH, C16. The representative plasma samples were pooled to produce qualitative control (QC) sample. 10 μL of QC sample and 90 μL of methanol was mixed and prepared as the described method in the section “Sample preparation” except ethanol/acetyl chloride instead of 1-butanol/acetyl chloride. 10 μL of combined standard solution with different diluted concentration and 90 μL of mixed stable isotope labeled standard solution was mixed and prepared as the described method in the section “Sample preparation” except derivatives redissolved in above supernatant. The peak area ratio of each stable isotope standard from the serially diluted reference standard solution to that from the reference standard solution was plotted against the corresponding concentration, and the calibration curve was constructed by means of the least-squares method.

### Sample preparation

The mixture of 10 μL thawed plasma and 90 μL mixed stable isotope labeled standard solution (as internal standard) was vigorously vortexed for 1 min, kept for 20 min at − 20 °C, and centrifuged at 16000 rcf for 15 min at 4 °C. The supernatant (80 μL) was transferred to a new tube and evaporated under nitrogen flow at 45 °C. The dried residues were redissolved in 100 μL freshly prepared 1-butanol/acetyl chloride (9:1, *v*/v), and the resulting mixture was heated at 65 °C for 15 min to obtain the butyl esters of carnitine and acylcarnitines. The derivatives were evaporated to dryness under nitrogen flow at 45 °C, and redissolved in 60 μL acetonitrile/water (4:1, v/v). The solution was centrifuged at 16000 rcf for 15 min at 4 °C, and supernatants were subjected to LC/MS/MS analysis.

### LC-MS/MS analysis

Liquid chromatographic separation was performed on an Agilent 1260 Liquid Chromatography system, equipped with well plate autosampler and thermostatic column compartment, coupled to an Agilent 6410B Triple Quadrupole Mass Spectrometer. Agilent MassHunter software was used for data acquirement.

A Waters HSS T3 (3.0 × 100 mm, 3.5 μm) column was used for chromatographic separation. Eight microliter of derivatives was injected to the column. The sampler temperature was maintained at 4 °C. The column temperature was maintained at 40 °C. A gradient system of two mobile phases, 0.1% formic acid in water (A) and 0.1% formic acid in acetonitrile (B), was used to elute the derivatives from column at a flow rate of 0.35 mL/min. The gradient was as follows: 0 min, 50% B; 1 min, 50% B; 9 min, 80% B; 11 min, 100% B; 16.5 min, 100% B; 17 min, 50% B; 23 min, 50% B. The total run time was 23 min.

The analytes were ionized in positive ESI mode. Data were acquired in dynamic multiple reaction monitoring (Dynamic MRM) mode. Nitrogen (99.999%) was applied as drying gas, nebulizer gas, and collision gas. The temperature and flow rate of drying gas were 300 °C and 8 L/min, respectively. The pressure of nebulizer gas was 40 psi. The capillary voltage was 4000 V. The fragment and collision energy (CE) were optimized for carnitine and each acylcarnitine. The concentration of each acylcarnitine was calculated from the calibration curve of corresponding stable isotope labeled standard. The other acylcarnitines without corresponding calibration curves were quantified using the calibration curves of the stable isotope labeled standards with similar structures or retention times due to the similar mass spectral signal response factor [12].

### Statistical analysis

Paired Wilcoxon’s Signed Rank tests were used to compare the eGFR level before and after the treatment. Wilcoxon’s Signed Rank tests were used for continuous variables and chi-squared tests were employed for categorical variables to compare baseline characteristics between participants with eGFR< 60 or not. Spearman’s partial correlation coefficients were calculated to examine associations among acylcarnitines after adjustment for age and sex. R package heatmap.2 was used to construct colored blocks representing levels of correlations.

Log-transformation was used for the eGFR and eGFR ratio as the responses variable of the linear regression. The cross-sectional associations of individual metabolites (scaled to SD of 1) with baseline eGFR (log transformed) were calculated using multivariate linear regression. Model was adjusted for age and sex or not. The longitudinal associations of acylcarnitines with eGFR change (log transformed) were also assessed by multivariate linear regression. To account for multiple testing, *p* value was adjusted with Benjamini & Hochberg (BH) correction. Acylcarnitines were considered significant when fulfilled with BH correction in the adjusted model. Further, Akaike’s Information Criterion (AIC) and Bayesian Information Criterions (BIC) value were calculated and compared. Variables were chosen by AIC in a Stepwise Algorithm. In addition, short-, medium-, and long-chain acylcarnitines categories were calculated as Z scores of acylcarnitines of carbon chains ≤6, 7–12, and ≥ 14, respectively. 1-year change in a given metabolite was calculated as the inverse normal transformed(INT) difference between the year-1 raw value and the baseline raw value. [13] Associations of 1-year changes of single acylcarnitines and combined indexes with eGFR Change (T2/T1) (log transformed) were also assessed by multivariate linear regression.

The statistical analyses were conducted using R (version 3.1.1; http://www.r-project.org). Two-sided *P* value < 0.05 was considered statistically significant if unspecified.

## Results

### Workflow

**Fig. 1:**
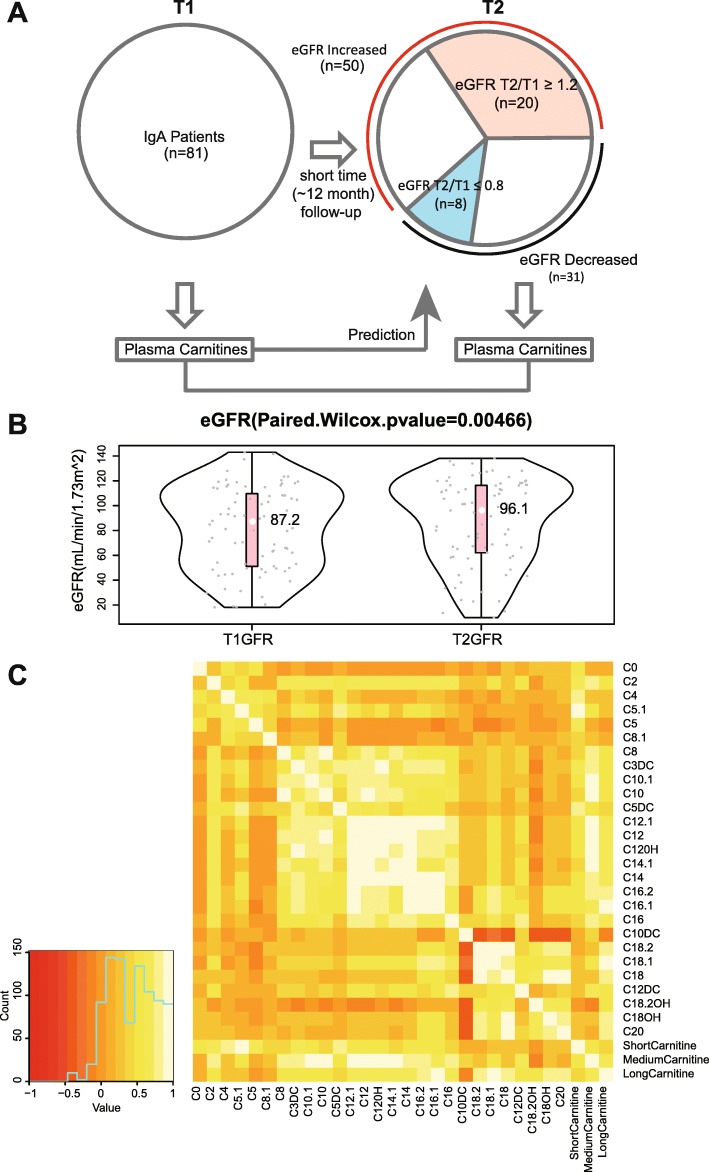
**a** Flowchart of IgAN patient treatment and measure of plasma acylcarnitines to predict prognosis and effect evaluation. **b** The eGFR could be increased after TCM-based treatment. **c** Correlations of acylcarnitines with different categories

As illustrated in the Fig. 1a, After the TCM-based treatment (range from 10 to 15 month, median = 12 month), 50(61.7%) participants showed increased eGFR level, among which 20(24.7%) increased more than 20%, 31(38.3%) participants showed decreased eGFR level, among which 8(9.9%) decreased more than 20%. The overall eGFR was improved significantly (Fig. 1b). The median eGFR was 87.2 mL/min/1.73m^2^ before treatment and increased to 96.1 mL/min/1.73m^2^ after treatment, with *p* value of 0.00466. For each patient, the plasma was collected before and after TCM-based treatment. Plasma acylcarnitines were quantified using liquid chromatography-tandem mass spectrometry (LC-MS/MS) for both T1 (before treatment) and T2 (after treatment) to predict and evaluate the effect of treatment (Fig. 1a).

### Baseline characteristics

**Fig. 2:**
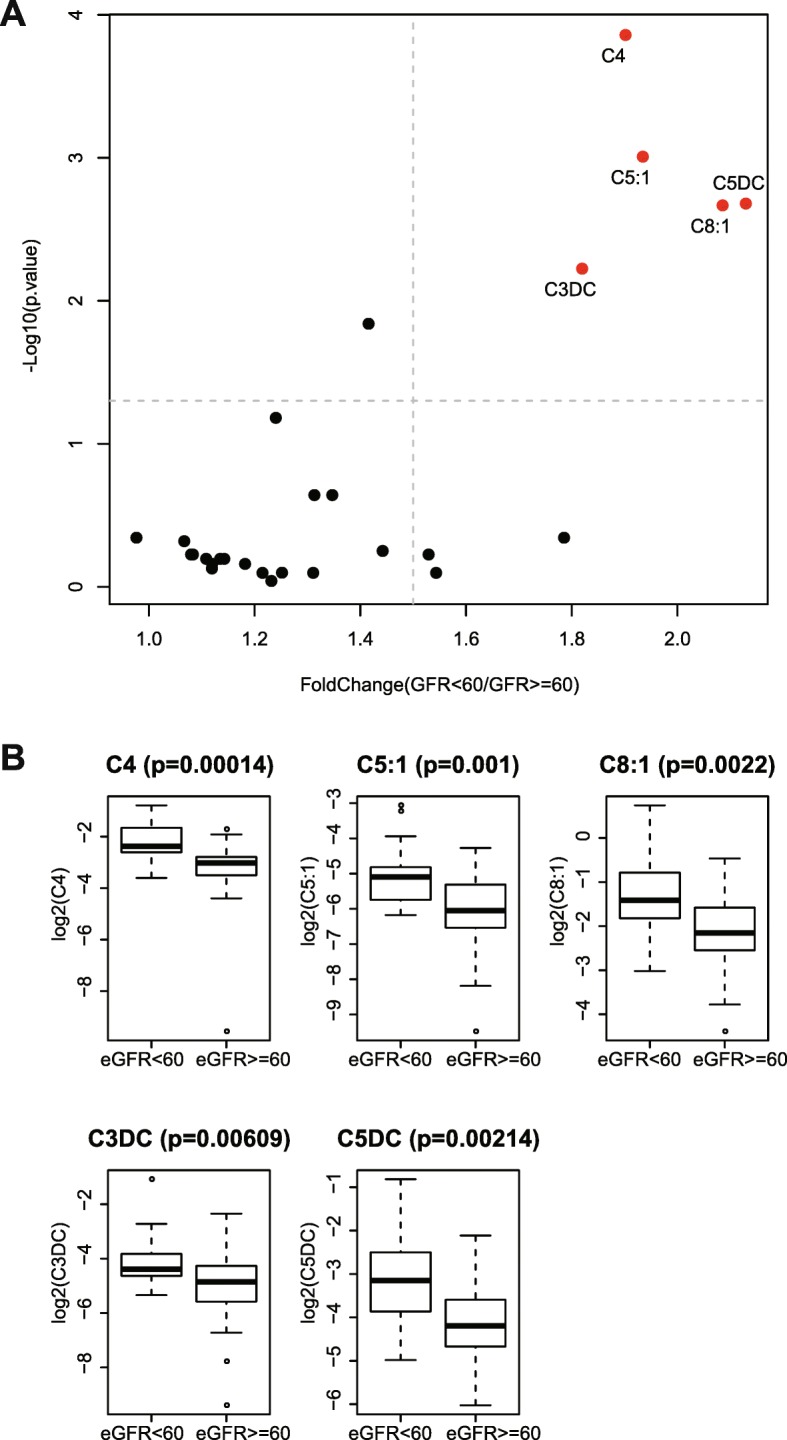
**a** Acylcarnitines comparison in baseline patients with eGFR< 60 and eGFR> = 60. Five Acylcarnitines (red dotted) showed increased in patients with eGFR< 60. **b** The differential concentrations of five acylcarnitines in baseline patients with eGFR< 60 and eGFR> = 60

The baseline characteristics of 81 participants were summarized in Table 1. Participants with lower eGFR values were older. They also exhibited higher levels of serum creatinine. The pairwise correlation structure of baseline plasma acylcarnitines was illustrated in Fig. 1c. Strong correlations were clustered within the same classes of acylcarnitines, like short-, medium- and long-chain acylcarnitines. We further stratified the patients to two groups with eGRF> = 60 or eGFR< 60, at T1 before treatment. Among the quantified acylcarnitines at baseline, 5 acylcarnitines including C4, C5:1, C8:1, C3DC, C5DC showed significantly higher concentrations in participants with lower eGFR values (Fig. 2a, b and Additional file 1: Table S1). The baseline characteristics of groups with increase of eGFR after treatment (*n* = 50) and decrease of eGFR (*n* = 31) showed no difference (Table 2).

**Table 1:** Baseline characteristics of the study

Characteristic	Total (*n* = 81)	Baseline eGFR< 60		
		Yes(*n* = 25)	No(*n* = 56)	*p*
Age, yr	41.8 ± 12.1	49.1 ± 11.8	38.6 ± 10.9	< 0.001
Male, n(%)	34(42.0)	8(32.0)	26(46.4)	0.331
Serum Creatinine, μmol/L	101.1 ± 56.0	159.4 ± 64.5	75.1 ± 22.5	< 0.001
Albumin, g/L	40.4 ± 5.0	38.5 ± 4.1	41.2 ± 5.1	0.010
24 h Proteinuria, g	1.3 ± 1.3	1.6 ± 1.4	1.2 ± 1.2	0.132
eGFR, mL/min/1.73m^2^	81.7 ± 33.4	40.7 ± 12.6	100.0 ± 21.2	< 0.001

Data are mean ± standard deviation, n(%)

**Table 2:** Baseline characteristics of groups with increase or decrease of eGFR after treatment

Characteristic	Total (*n* = 81)	T2T1GFRRatio > 1		
		Yes(*n* = 50)	No(*n* = 31)	*p*
Age, yr	41.8 ± 12.1	41.3 ± 11.1	42.6 ± 13.8	0.815
Male, n(%)	34(42.0)	24(48.0)	10(32.3)	0.244
Serum Creatinine, μmol/L	101.1 ± 56.0	100.2 ± 47.9	102.5 ± 67.9	0.431
Albumin, g/L	40.4 ± 5.0	40.8 ± 5.3	39.7 ± 4.4	0.288
24 h Proteinuria, g	1.3 ± 1.3	1.2 ± 1.3	1.5 ± 1.3	0.133
eGFR, mL/min/1.73m^2^	81.7 ± 33.4	81.6 ± 30.4	82.0 ± 38.3	0.760

Data are mean ± standard deviation, n(%)

### Cross-sectional association between metabolites and eGFR at baseline

In cross-sectional analysis at baseline, 12 acylcarnitines including C2, C4, C5:1, C5, C8:1, C3DC, C10:1, C5DC, C12:1, C12OH, C16:2, C16:1 were significantly negatively associated with baseline eGFR, after adjusting for age and sex. When acylcarnitines were classified as categories, only short-chain and medium-chain species, but not those long-chain ones, were significantly associated with baseline eGFR (Table 3).

**Table 3:** Associations of single acylcarnitines and combined indexes with baseline eGFR

Name	Association with baseline eGFR		
	β	SE	*P*
Free carnitine (C0)	−0.21	0.11	0.110108011
Acetylcarnitine (C2)	−0.36	0.1	0.001989281
Butyrylcarnitine (C4)	−0.63	0.08	7.42E-10
semi Tiglyl-carnitine (C5:1)	−0.56	0.09	4.10E-07
Valerylcarnitine (C5)	−0.37	0.11	0.002867891
semi Octanenoylcarnitine (C8:1)	−0.46	0.1	0.00012474
Octanoylcarnitine (C8)	−0.13	0.11	0.333735122
Malonylcarnitine (C3DC)	−0.41	0.1	0.000543281
C10:1	−0.32	0.11	0.011733593
Decanoylcarnitine (C10)	−0.12	0.11	0.336089636
C5DC	−0.65	0.08	3.01E-10
semi Dodecenoylcarnitine (C12:1)	−0.25	0.1	0.041281611
Dodecanoylcarnitine (C12)	−0.19	0.11	0.147639204
semi 3-Hydroxydodecanoylcarnitne (C12OH)	−0.27	0.11	0.03221931
C14:1	−0.16	0.11	0.227662143
Myristoylcarnitine (C14)	−0.23	0.1	0.061749229
semi (C16:2)	−0.26	0.1	0.035592205
semi Palmitoleylcarnitine (C16:1)	− 0.25	0.1	0.04354928
Palmitoylcarnitine (C16)	−0.16	0.11	0.227662143
semi Sebacylcarnitine (C10DC)	−0.12	0.11	0.366303947
semi Linoleylcarnitine(C18:2)	−0.06	0.11	0.641083979
semi Oleylcarnitine (C18:1)	−0.06	0.11	0.641083979
semi Stearoylcarnitine (C18)	−0.11	0.11	0.410468226
semi C12DC	−0.12	0.11	0.353100378
semi 3-Hydroxylinoleyl(C18:2OH)	0.05	0.11	0.682991111
semi 3-Hydroxystearoylcarnitine (C18OH)	−0.08	0.11	0.53159956
semi Arachidoylcarnitine (C20)	−0.13	0.11	0.333735122
Short-chain Acylcarnitine	−0.13	0.05	0.028647383
Medium-chain Acylcarnitine	−7.66	1.88	0.000543281
Long-chain Acylcarnitine	−0.19	0.77	0.800766318

*β* effect size, *SE* standard error

Model was adjusted for age and sex

*P* value was adjusted with BH correction

### Baseline acylcarnitines could predict eGFR change by treatment

eGFR ratio (T2-treatment/T1-baseline) was used to evaluate the effect of TCM-based treatment. As a result, 11 out of 27 baseline acylcarnitines were significantly associated with eGFR change, after adjusting for age, sex. When additionally controlling for baseline eGFR, as many as 15 out of 27 acylcarnitines achieved Benjamini & Hochberg (BH)-level significance for eGFR change, including C2, C5:1, C8:1, C3DC, C10:1, C10, C5DC, C12:1, C12, C12OH, C14:1, C14, C16:2, C16:1, C16 and the *P* value of 11 acylcarnitines were smaller than 0.01 (Table 4, Additional file 2: Table S2, Model 4). Especially, the baseline medium-chain acylcarnitine categories (*P* = 1.09e-5) were significantly associated with eGFR change after 1 year, after adjusting for baseline eGFR, age and sex. Moreover, 5 out of 15 acylcarnitines, that were C5:1, C8:1, C3DC, C10:1 and C5DC, passed variable selection and formed a predictive model (Additional file 2: Table 2, model 3). C5DC was with the smallest adjusted *p* value and the largest effect size per SD (β = − 0.64) (Additional file 2: Table 2, model 2). Compared with the conventional model (Additional file 2: Table 2, model1), model3 have the smallest AIC and BIC value (AIC = 179.8485, BIC = 203.793, *P* = 6.914E-10) (Additional file 2: Table 2), which is the most accurate prediction.

**Table 4:** Associations of single acylcarnitines and combined indexes with short-time eGFR Change(T2/T1)

Name	Total(*n* = 81)(adjust for age and sex)			Total(*n* = 81)(adjust for age, sex and baseline eGFR)		
	β	SE	P	β	SE	P
Free carnitine (C0)	0.15	0.11	0.245907215	0.12	0.12	0.350241611
Acetylcarnitine (C2)	− 0.21	0.1	0.089140039	−0.29	0.11	0.01961784
Butyrylcarnitine (C4)	0.01	0.11	0.941984236	−0.12	0.13	0.411285924
semi Tiglyl-carnitine (C5:1)	−0.24	0.11	0.066284748	−0.42	0.12	0.002225674
Valerylcarnitine (C5)	−0.01	0.11	0.941984236	−0.07	0.12	0.619468149
semi Octanenoylcarnitine (C8:1)	−0.35	0.11	0.005973723	−0.49	0.11	0.000191111
Octanoylcarnitine (C8)	−0.22	0.1	0.083296427	−0.23	0.1	0.050106168
Malonylcarnitine (C3DC)	−0.39	0.1	0.003217745	−0.5	0.1	5.30E-05
C10:1	−0.29	0.11	0.019670285	−0.36	0.11	0.003423257
Decanoylcarnitine (C10)	−0.24	0.1	0.053100727	−0.26	0.1	0.029386435
C5DC	−0.34	0.1	0.005973723	−0.64	0.11	6.34E-06
semi Dodecenoylcarnitine (C12:1)	−0.36	0.1	0.004133772	−0.4	0.1	0.000707745
Dodecanoylcarnitine (C12)	−0.31	0.1	0.011483585	−0.34	0.1	0.00408751
semi 3-Hydroxydodecanoylcarnitne (C12OH)	−0.34	0.1	0.005973723	−0.39	0.1	0.001150804
C14:1	−0.27	0.1	0.024970382	−0.3	0.1	0.011921285
Myristoylcarnitine (C14)	−0.36	0.1	0.004133772	−0.4	0.1	0.000707745
semi (C16:2)	−0.35	0.1	0.004509645	−0.39	0.1	0.000760176
semi Palmitoleylcarnitine (C16:1)	−0.35	0.1	0.004509645	−0.39	0.1	0.000760176
Palmitoylcarnitine (C16)	−0.22	0.11	0.083296427	−0.24	0.11	0.045134394
semi Sebacylcarnitine (C10DC)	−0.14	0.11	0.266164949	−0.16	0.11	0.195395601
semi Linoleylcarnitine(C18:2)	−0.15	0.11	0.245907215	−0.16	0.11	0.21529555
semi Oleylcarnitine (C18:1)	−0.18	0.11	0.171164932	−0.18	0.11	0.152392876
semi Stearoylcarnitine (C18)	−0.15	0.11	0.245907215	−0.16	0.11	0.195395601
semi C12DC	−0.21	0.11	0.099705511	−0.22	0.11	0.070188377
semi 3-Hydroxylinoleyl(C18:2OH)	−0.02	0.11	0.941984236	−0.01	0.11	0.930007953
semi 3-Hydroxystearoylcarnitine (C18OH)	−0.04	0.11	0.840418859	−0.05	0.11	0.711962011
semi Arachidoylcarnitine (C20)	−0.15	0.11	0.245907215	−0.17	0.11	0.195395601
Short-chain Acylcarnitine	0.04	0.05	0.537548196	0.02	0.05	0.761254671
Medium-chain Acylcarnitine	−7.83	1.87	0.002164496	−10.12	1.87	1.09E-05
Long-chain Acylcarnitine	−0.86	0.76	0.314539124	−0.87	0.76	0.313630234

*β* effect size, *SE* standard error

*P* value was adjusted with BH correction

**Fig. 3:**
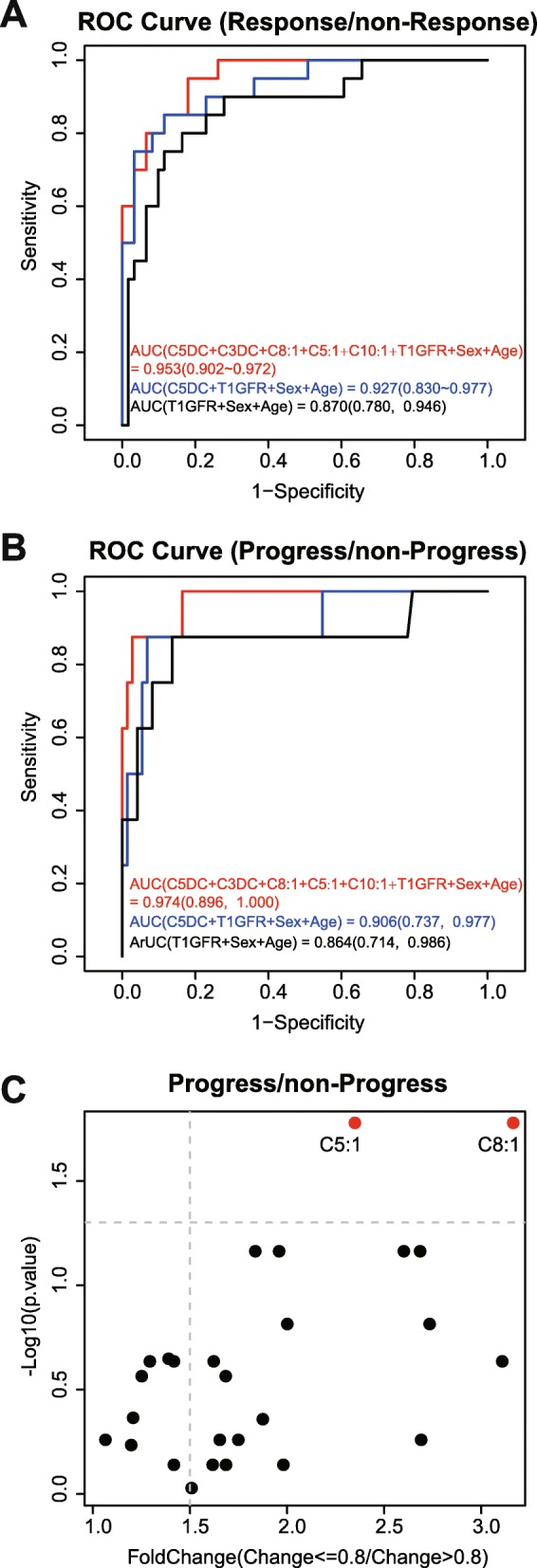
**a** ROC curve using baseline characteristics to predict treatment response (eGFR T2/T1 > =1.2) and non-response (eGFR T2/T1 < 1.2). **b** ROC curve using baseline characteristics to predict disease progress (eGFR T2/T1 < =0.8) and non-progress (eGFR T2/T1 > 0.8). **c** Baseline acyl-carnitines comparison in patients with progress (eGFR T2/T1 < =0.8) and non-progress (eGFR T2/T1 > 0.8)

Further, to explore the predictive ability of established models, we classified the participants into 2 groups: response and non-response group according the criteria of eGFR ratio (T2-treatment/T1-baseline) ≥1.2 or not and performed Receiver operating characteristic (ROC) curve analyses. (Fig. 3a) The AUC was 0.870 in the conventional model 1 and significantly improved to 0.953 in the model 3 with acylcarnitines C5:1, C8:1, C3DC, C10:1 and C5DC as variables. Just using C5DC in the model 2, the AUC was 0.927, also bigger than the conventional model. Also,a leave-one-out cross-validation was used. The AUC was 0.816 in the conventional model and improved to 0.864 in the acylcarnitine-added model. The most frequently-selected features also included C5:1, C8:1, C3DC, C10:1 and C5DC, which verified the features selected by regression model. If we changed the classification criteria: eGFR ratio (T2-treatment/T1-baseline) ≤0.8 or not and classified the participants into progress and non-progress group, the conclusion was same (Fig. 3b). Moreover, C5:1 and C8:1 showed significant differences in progress and non-progress group, but not in response and non-response group (Fig. 3c).

### One-year change in acylcarnitines and the effect of TCM-based treatment

**Fig. 4:**
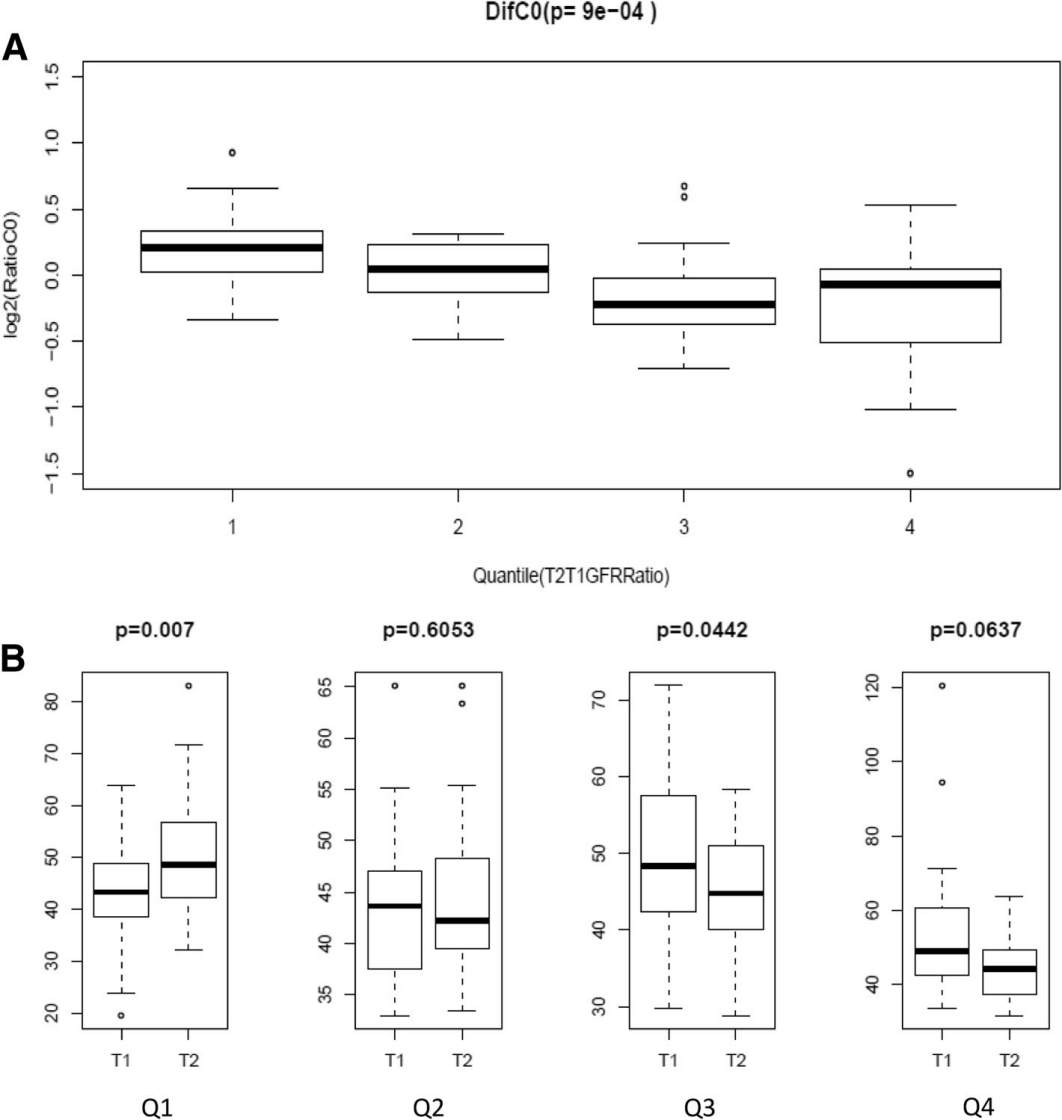
**a** C0 carnitine concentrations ratios (T2/T1) are significantly different in quantiles according to eGFR ratios (T2/T1). **b** C0 carnitine concentrations before and after treatment in each quantiles of eGFR changes (T2/T1)

Associations of 1-year changes of single acylcarnitines and combined indexes with eGFR changes were also calculated. As results, C0, C4, C5, C8:1 and C3DC were with significantly negative association with eGFR change (Table 5). According to quantile of the ration of eGFR after and before treatment (T2/T1), C0 showed significant different among the quantiles of eGFR changes (Fig. 4a, *p* = 9e-04). In addition, C0 showed significant increase after treatment in the bottom quantile with decrease of T2/T1 eGFR ratio (*P* = 0.007). However, for the patient with some increase of eGFR (Q3 and Q4), C0 showed somehow decrease (Fig. 4b).

**Table 5:** Associations of 1-year INT changes of single acylcarnitines(Dif) and combined indexes with eGFR Change(T2/T1)(Log2 transformed)

Log2(eGFR Change),INT(CarDif)	Adjust T1GFR + T1Carnitine + Age + Sex		
	β	SE	P
Free carnitine (C0)	−0.49	0.13	0.007492944
Acetylcarnitine (C2)	−0.34	0.13	0.06034281
Butyrylcarnitine (C4)	−0.43	0.12	0.008221232
semi Tiglyl-carnitine (C5:1)	−0.12	0.12	0.599218799
Valerylcarnitine (C5)	−0.37	0.14	0.042476427
semi Octanenoylcarnitine (C8:1)	−0.29	0.11	0.042476427
Octanoylcarnitine (C8)	0.02	0.13	0.981932317
Malonylcarnitine (C3DC)	−0.37	0.1	0.008221232
C10:1	−0.11	0.14	0.70587576
Decanoylcarnitine (C10)	0.02	0.13	0.981932317
C5DC	−0.21	0.09	0.087001606
semi Dodecenoylcarnitine (C12:1)	−0.17	0.12	0.42430295
Dodecanoylcarnitine (C12)	−0.01	0.13	0.981932317
semi 3-Hydroxydodecanoylcarnitne (C12OH)	−0.15	0.12	0.513747479
C14:1	0	0.14	0.981932317
Myristoylcarnitine (C14)	0.01	0.12	0.981932317
semi (C16:2)	−0.17	0.12	0.42430295
semi Palmitoleylcarnitine (C16:1)	−0.16	0.12	0.426208624
Palmitoylcarnitine (C16)	−0.11	0.12	0.645845281
semi Sebacylcarnitine (C10DC)	−0.09	0.13	0.732411215
semi Linoleylcarnitine(C18:2)	0.05	0.12	0.934179803
semi Oleylcarnitine (C18:1)	−0.03	0.12	0.981932317
semi Stearoylcarnitine (C18)	−0.07	0.13	0.870583478
semi C12DC	−0.11	0.13	0.70587576
semi 3-Hydroxylinoleyl(C18:2OH)	−0.02	0.14	0.981932317
semi Arachidoylcarnitine (C20)	−0.19	0.13	0.42430295

*β* effect size, *SE* standard error

*P* value was adjusted with BH correction

## Discussion

Our work demonstrated that plasma acylcarnitines in IgA patients are associated with prognosis and treatment effect. The baseline levels of acylcarnitines were related to 1-year eGFR changes, in which five acylcarnitines including C5:1, C8:1, C3DC, C10:1 and C5DC could predict 1-year renal function. The 1-year acylcarnitines also changed along with the trends of eGFR after treatment.

Previous acylcarnitines data related to kidney function are limited, while the results about therapy effect are rare. Replicated associations with eGFR were observed for 22 metabolites in 2 separate population studies by Goek et al. [14], 12 of which were measured in our study. 7 carnitines were found to be significantly and consistently associated with eGFR in the same direction in our data. For example, glutarylcarnitine(C5DC) was negatively associated with eGFR, having the lowest *P* value in both study. Of 4 overlap carnitines reported by Yu B et al. [15], tiglylcarnitine were concordant with our findings. Interestingly, free carnitine was found to be negatively associated with eGFR in the study by Goek et al. [14], positively associated with eGFR in the study by Yu B et al. [15], but not significantly associated with eGFR in our study. One possible reason is the difference of population characteristics. The participants their studies were from general population or individuals with normal kidney function. The participants here were all IgAN patients with impaired kidney function, which will influence the production, conversion, and clearance of carnitine. Meanwhile, the cross-ethnical difference in genetic background and dietary exposure might contribute to the discrepancies of the studies.

We recently reported the association of acylcarnitine with risk of diabetes in six years [12]. The aclycarnitines could also predict the Cardiovascular disease (CVD) events [16, 17]. However in the CVD or type 2 diabetes (T2D) associated acylcarnitines, short-chain (C2-C7) and long-chain (C16-C26) forms seem more significant [16, 17]. In our study, most of baseline medium-chain (C8-C16) acylcarnitines showed obviously negative association with 1-year eGFR change after treatment. In short-chain group, C2, C3DC, C5:1 and C5DC demonstrated significant association with 1-year eGFR alteration. These observations indicated the role of acylcarnitines might be different in renal function with CVD and T2D. The acylcarnitines are involved with lipid metabolism, amino-acid metabolism, inflammation process as well as mitochondria function [18–20]. The long-chain acylcarnitines are involved with fatty acid metabolism, while short- or median-chain acylcarnitines might be more associated with amino-acid metabolism. Our study indicates that CKD could have more dysregulation related to amino-acid metabolism or protein nutrition.

Our previous work presented the effect of TCM to protect renal function in IgA and membranous nephropathy (MN) patients [8, 9, 21]. Currently, though corticosteroids-based therapy could result in slower rate of renal function decline, the overall eGFR was still declined [22]. In this study, with TCM-based treatment, the overall eGFR could be improved or maintained, thus supporting the effect TCM-based treatment for IgAN [8, 9]. The accompanied measurement of certain biomarkers could facilitate the stratification of patients and result in more precise treatment. In this work, the baseline acylcarnitines are negatively associated with eGFR change after 1 year treatment, which could be used to predict patient prognosis and treatment response. Particularly, patients with high C5:1, C8:1, C3DC, C10:1 and C5DC at baseline would have worse prognosis and treatment response. The previous work has reported that acylcartinines could be regulated by dietary intervention [16]. We also found certain acylcarnitines (C0, C4, C5, C8:1 and C3DC) changes would be related to eGFR alterations by treatment. Impaired kidney function may affect the level of a wide range of serum metabolites, including acylcarnitines. Meanwhile, metabolites may also contribute to CKD progression and therapy effect. These observations indicate that acylcarnitines could be molecular responders or markers for intervention, regulated by either nutritional condition or drug treatment.

The main limitation of the present work is that the population is small and lack of further follow-up data. It is also unclear that the association of acylcarnitines with eGFR could be useful for long-term prediction of renal functions. As an observation study, some of parameters such as BMI, UACR and blood pressure are missing. Further prospective study should be performed to confirm the observations in this work. On the other hand, more investigations should be performed in other CKD patients, in order to determine specific markers for different renal diseases.

## Conclusions

The present work demonstrated the association of plasma acylcarnitines with renal function and its alteration by intervention. These observations provided the basis to predict prognosis and evaluate treatment effect using metabolic profiling.

## Additional files

Additional file 1: **Table S1.** Characteristics of the Baseline Plasma Acylcarnitines. (XLSX 13 kb)

Additional file 2: **Table S2.** Predict models. (XLSX 9 kb)

## Abbreviations

AIC: Akaike’s Information Criterion
BH: Benjamini& Hochberg
BIC: Bayesian Information Criterions
CE: Collision energy
CKD: Chronic kidney disease
CVD: Cardiovascular disease
EDTA: Ethylene diamine tetraacetic acid
eGFR: Estimated glomerular filtration rate
HBV: Hepatitis B virus
IgAN: IgA nephropathy
INT: Inverse Normal Transformation
LC-MS/MS: Liquid chromatography-tandem mass spectrometry
MDRD: Modification of Diet in Renal Disease
MN: membranous nephropathy
MRM: Multiple reaction monitoring
QC: Qualitative control
ROC: Receiver operating characteristic
T2D: type 2 diabetes
TCM: Traditional Chinese medicine
